# An ethnozoological study in the adjoining areas of Mount Abu wildlife sanctuary, India

**DOI:** 10.1186/1746-4269-6-6

**Published:** 2010-02-10

**Authors:** DP Jaroli, Madan Mohan Mahawar, Nitin Vyas

**Affiliations:** 1 Department of Zoology, University of Rajasthan, Jaipur (Rajasthan), India; 2 Department of Zoology, Govt. P.G. College, Sawai Madhopur (Rajasthan), India

## Abstract

**Background:**

There is evidence that human beings are familiar with use of animals for food, cloth, medicine, etc. since ancient times. Enormous work has been done on ethnobotany and traditional medicine. Like plants, animal and their products are also possessing medicinal properties that can be exploited for the benefit of human beings. In India, many ethnic communities are dispersed all over the country and these people are still totally depended on local traditional medicinal system for their health care. India is gifted with faunal and floral biodiversity, Mount Abu wildlife sanctuary is also one of them, and thus the aim of this work was to take an ethnozoological field survey among Garasiya people (main tribal group of this area) in the adjoining areas of this sanctuary.

**Method:**

In order to document the ethnozoological information about animal and their products prevalent among these people in the adjoining area of Mount Abu wildlife sanctuary, a study was carried out from January, 2008 to April, 2008. Data were collected through semi-structured questionnaire and open interview with 25 (16 male and 9 female) selected Garasiya people. The name of animal and other ethnozoological information were documented. Photographs and discussion were also recorded with the help of camera and voice recorder.

**Result:**

A total of 24 animal species were used in 35 different medicinal purposes including asthma, weakness, tuberculosis, cough, paralysis and blister and for other religious purposes. It has been find out that animal used by Garasiya, consist of fourteen mammals, five birds, three reptiles, one arthropods and one amphibian. The meat of *Cynopterus sphinx *used to relieved fever and cough has the highest FL (96%) although flesh of *Sus scrofa *and tooth of *Elephas maximus *have the lowest FL (12%). Some protected species such as *Elephas maximus *(elephant), *Semnopithecus priam *(monkey), *Cervus unicolor *(sambhar) were also mentioned as important medicinal resources. We also found that cough, asthma and other respiratory diseases are the most frequently cited disease, as such, a number of traditional medicine are available for the treatment.

**Conclusion:**

The present work indicates that 24 animal species were being used to treat 34 various ailments in the surroundings areas of Mount Abu wildlife sanctuary. The results show that ethnozoological practices are an important alternative medicinal practice for the Garasiya people. This study also indicates the very rich ethnozoological knowledge of these people in relation to traditional medicine. So there is an urgent need to properly document to keep a record of the ethnozoological information. We hope that this information will be useful for further research in the field of ethnozoology, ethnopharmacology and conservation point of view.

## Background

There is evidence that human beings are familiar with use of animals and plants for food, cloth, medicine, etc. since ancient times [1]. Ethnozoology deals with the study of relationship between the human societies and the animal resources around them [2]. Zootherapy is an important component of ethnozoology, the healing of human ailments by using therapeutic based on medicine obtained from animals or ultimately derived from them in known as zootherapy [3]. The Zootherapeutic resources constitute the essential ingredients in different traditional systems [4].

Since ancient time's animals, their parts, and their products have constituted part of the inventory of medicinal substances used in various cultures [5]. The world health organization estimates that as many as 80% of the world's population (more then six billion people) rely primarily on animal and plant based medicines [6]. In Traditional Chinese Medicine more then 1500 animal species have been recorded to be some medicinal use [7]. Of the 252 essential chemicals that have been selected by the World Health Organization, 8.7% come from animals [8]. Alves and Rosa recorded the use of 97 animal species as traditional medicine in urban areas of NE and N Brazil [9]. Lev and Amar conducted a survey in the selected markets of Israel and found 20 animal species, which products were sold as traditional drugs [10]. In Brazil, Alves et al. reported the medicinal use of 283 animal species for the treatment of various ailments [11]. In Bahia state, in the northeast of Brazil, over 180 medicinal animals have been recorded in traditional health care practices [12]. 11 animal species were identified, which by-products were used in zootherapeutic purposes by Tamang people of Nepal [13]. Alves and Rosa carried out a survey in fishing communities located in the North and north-east regions of Brazil and recorded 138 animal species, used as traditional medicine [14]. Lev and Amar conducted a study in the selected markets in the kingdom of Jordan and identified 30 animal species, which products were sold as traditional drugs [15]. Alves et al. also reported that at least 165 reptile's species were used in traditional folk medicine around the world [16]. Alves conducted a review study in Northeast Brazil and inventories 250 animal species for the treatment of different ailments [17].

In India, since times immemorial, great work was done in the field of zootherapy, traditional medicine and documented in works like *Ayurveda *and *charaka Samhita*. A number of animals are mentioned in *Ayurvedic *system, which includes 24 Insects, 16 Reptiles, 21 Fishes, 41 Aves and 41 Mammals [18]. Different ethnic group and tribal people use animals and their products for healing practices of human ailments in present times in India. The Hindu religion has used five products (milk, urine, dung, curd and ghee) of the cow for purification since ancient times [19]. Gupta et al. describe the traditional knowledge of local communities in district Kachchh, Gujarat and identified 34 animals and bird species, which were used in primary health care of human beings and livestock [20]. Patil found that the tribals of Maharashtra have been use wild animal parts as medicines along with plants. This study assesses 15 species of animals used by the tribal like *Bhils, Gamits, Koknas *and *Pawaras *as traditional medicinal resources [21]. Jamir and Lal describe the traditional method of treating various kinds of ailments using twenty six animal species and their products by different Naga tribes [22]. 16 animal species were identified for the treatment of over 17 kinds of ailments in Tamilnadu [23]. 15 animal species were recorded comprising 20 therapeutic purposes in surroundings area of the Ranthambhore National Park (RNP) India [24]. A total of 38 animal species, belonging to 16 families were either being used by the common-folk in the treatment of various ailments or were in possession of the knowledge base of the usage of the same by *shoka *tribe of Uttaranchal, India [25]. Dash and Pandhy discussed that various components of the human body like blood, bone, hair etc. were also used as directly or indirectly against diseases [26]. Solanki and Chutia carried out an ethnozoological study of Arunachal Pradesh, India and identified various animal species used in traditional medicinal system [27]. 44 animal species and their products were identified from Attappady hills of Western Ghats, India which were used by *irular*, *mudugar *and *kurumbar *tribal people [28]. 15 animal species were recorded used for different ethnomedicinal purpose among *saharia *tribes of Rajasthan, India [29]. Jain et al. carried out an ethnomedicinal survey among the different ethnic groups (*Bhil, Meena, and Garasia*) of Tadgarh-Raoli wild life sanctuary, India and identified several substances of animal origin to relieve various ailments through indigenous health care practices [30]. Mahawar and Jaroli carried out a review study and identified 109 animal species and their 270 uses in traditional medicine in different parts of India [31].

India is gifted with immense faunal and floral biodiversity, because of the extreme variation in geographical and climatic condition prevailing in the country. There are about 45000 species of plants and 81000 species of animals [32]. In India, different tribal and ethnic communities are dispersed all over the country, people of these communities are highly knowledgeable about the animals and their medicinal value, and they also provide considerable information about the use of animals and their by-products as medicine. Most of the rural areas, tribal and ethnic people are totally dependent on local traditional medicinal system for their health care because they are living in remote areas where hospital and other modern medicinal facilities are not available, so they use their traditional knowledge for medicinal purpose and this knowledge is passed through oral communication from generation to generation.

Enormous work has been done on utilization of plants and their products as traditional and allopathic medicine. Like plants, animal and their products also possess medicinal properties [33]. A lot of work has been done in the Mount Abu wildlife sanctuary on the ethnobotany and medicinal plants and documented too, but there is a definite scarcity of ethnobiological knowledge when it comes to animal products. Only few previous workers were carried an ethnomedicinal study among Garasiya people, such as, S. K. Sharma [34], and Jain et al. carried an ethnomedicinal study among Garasiya people in Tadgarh-Raoli Wildlife Sanctuary, India and identified several animal species used in traditional medicine [30]. The present study briefly reports the use traditional medicine of animal origin by Garasiya people of Rajasthan.

## Methods

### The study area

**Figure 1:**
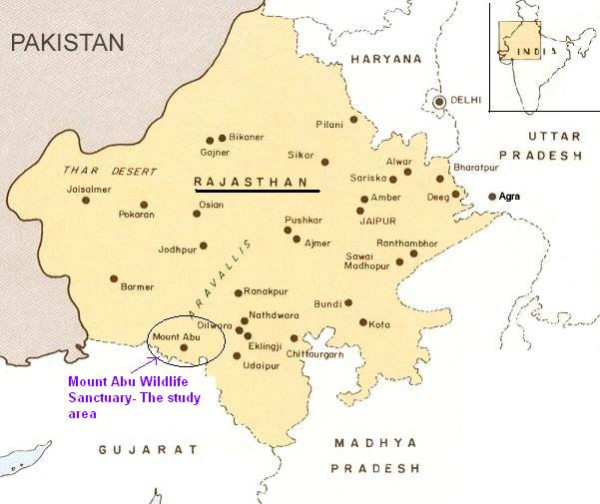
**Map of the study area**.

The Mount Abu wildlife sanctuary is located in the Southwestern Rajasthan close to the border of Gujarat state of India, in Sirohi district of Rajasthan. The sanctuary comprises oldest Mountain range of Aravalli hills; it was declared a wildlife sanctuary in 1960. The sanctuary spread over 288 kms and is located between 24°33' and 24°43' north latitude and 72°38' and 72°53' east longitude. It is 300 m to 1722 m high from sea level. The climate of Mount Abu varies from the foot hills to high altitude, it is hot and dry at the base while pleasant and moderate at the top for the greater part of the year. The summer (March to July) temperature of the sanctuary varies between 23 to 35°C and in winter (November to February) it is between -2 to 25°C with 150 cm average rainfall. The sanctuary exhibit a great ethnic, cultural, floral and faunal diversity, it is a very popular destination for eco-tourism. A variety of fauna including highly rare and endangered species are found in this sanctuary. The past history of the sanctuary indicates the presence of lions and tigers. There are over more than 200 species of bird including the popular grey jungle fowl, it also houses of panther, leopard, sloth bear, sambhar, chinkara, jackal, chameleon, elephant, deer, wild dog and languor amongst others. The ethnozoological study was mainly conducted in the village's surroundings the sanctuary. The Garasiya are the main tribal group which lives around the sanctuary, so most of these data were collected from Garasiya people [Figure 1].

### The Garasiya tribe

**Figure 2:**
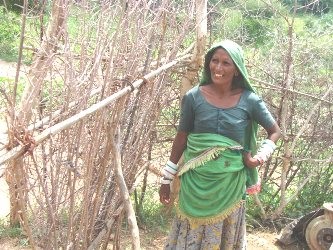
**Picture of *Garasiya *women (Photo: Nitin Vyas)**.

The Garasiya people are main inhabitant of surroundings areas of the Mount Abu wildlife sanctuary, Pindwara and Aburoad *tehsil *of Sirohi district of Rajasthan. Earlier they would live under rock shades and caves, which provided shelter to these people. They are traditionally a nomadic community and speak *Marwari *and *Godwari *as their languages. Garasiya are generally thin, short in height and balanced body, woman are generally shy, honest and laborious. They are very co-operative in nature and peace loving people. The economic condition of the Garasiya is not good. Agriculture, animal husbandry; poultry forming and laboring are source of income. Occasionally they are indulged in robbery and theft due to poverty. They also collect gum, traditional medicine and honey and sale to generate income. The life of the people are full of traditions and social customs from birth to death owning to outdated customs, not attuned to remain competitive in the current economic scenario of privatization. The birth of the male child is given more importance than the female child. Polygamy and *Dapa *(tradition of marriage practice in which in-laws of the girls give money to the in-laws of the boys) are common practice among these people. Due to living in remote areas, traditional culture, large number of family member and poverty their children are not able to take even primary education, only 5-10% children get primary education, higher education and girls education being negligible. The Garasiya people residing in the remote hilly and deep forest areas still dependent on plants and animals for their primary health care and for treatment for various ailments [Figure 2].

### Procedures

In order to acquire ethnozoological information about animal and their products used in traditional medicine, a study was conducted from January, 2008 to April, 2008 in the adjoining areas of the Mount Abu wildlife sanctuary, India. The ethnomedicinal data (local name of animals, mode of preparation and administration) were collected through semi-structured questionnaire (in their local language, with the help of local mediator), interview and group discussion with selected Garasiya people. The selection of informants was based on their experience, recognition as expert and knowledge old aged person concerning traditional medicine. A total of 25 (16 male and 9 female) people were selected to collect ethnozoological information, these information were collected from local herbalist, healers, farmers and local doctor. We interviewed 16 (64%) informants within age group 55 and above, followed by 06 informants (24%) with 45 to 54 age group and 03 (12%) with 35-44 years age group.

They were asked, about the ailments cured by animal based medicines and the manner in which the medicines were prepared and administered. They were also asked detailed information about mode of preparation and blending of animal products used as ingredients and whether they use animal in the healing practice, since this kind of information indicate how a given medicine can be therapeutically efficient in term of the right ingredients, the proper dose and the right length of medication. The name of animals and other information related to this study were documented. Some photographs of Garasiya people at their local place and in their traditional life style in study area were taken; discussion was also recorded with the help of voice recorder.

According to them, their traditional ethnozoological knowledge was mainly acquired through parental heritage and experience about medicinal value of animal to heal their kin or themselves. The scientific name and species of animals were identified using relevant and standard literature [35,36].

### Data analysis

For the data analysis, fidelity level (FL) calculated that demonstrates the percentage of respondents claiming the use of a certain animal species for the same ailments, was calculated for the most frequently reported diseases or ailments as:

Where Np is the number of respondents that claim a use of a species to treat a particular disease, and N is the number of respondents that use the animals as a medicine to treat any given disease [37]. The range of fidelity level (FL) is from 1% to 100%. high use value (close to 100%) show that this particular animal species are used by large number of people while a low value show that the respondents disagree on that spices to be used in the treatment of ailments.

## Result and Discussion

The present study revealed the traditional medicinal knowledge of treating various kinds of ailments using different animal and their products by the local Garasiya people inhabitants of villages in the adjoining areas of the Mount Abu wildlife sanctuary, India. Many people were found to lack formal schooling education, but they have knowledge about use of local animal and plant resources for traditional medicinal and other religious purposes [13], Garasiya people are one of them [Additional file 1].

**Figure 3:**
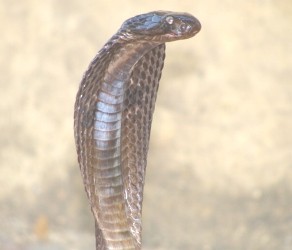
**Picture of *Naja naja***.

**Figure 4:**
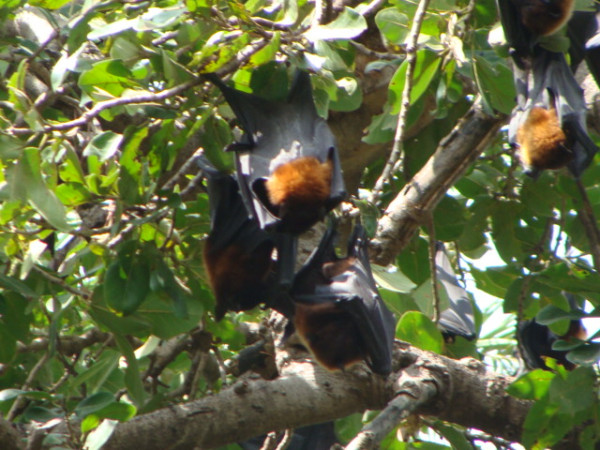
**Picture of *Cynopterus sphinx***.

**Figure 5:**
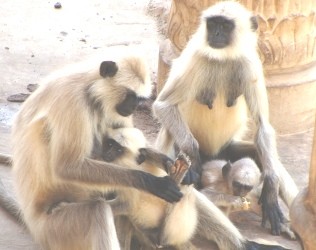
**Picture of *Semnopithecus entellus***.

**Figure 6:**
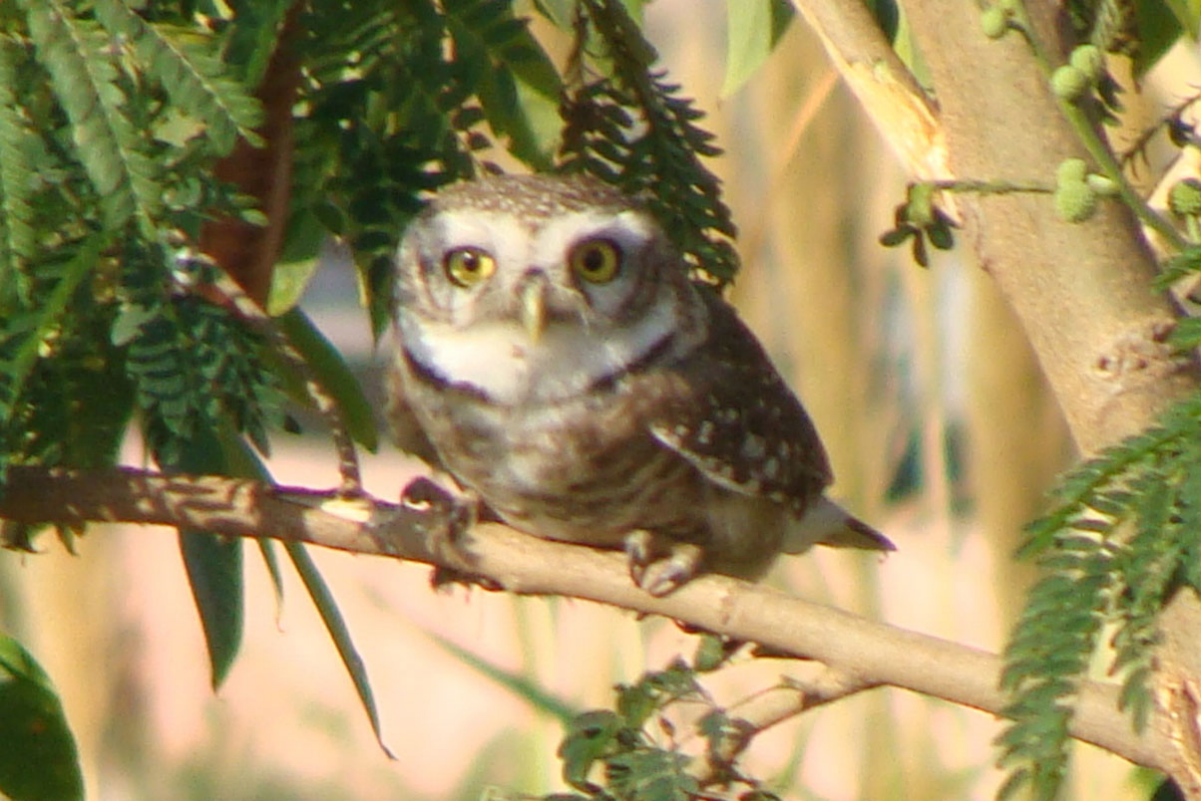
**Picture of *Otus bakkamoena *(Photos: Nitin Vyas)**.

Additional file 1 shows that, *Garasiya *people of Rajasthan were using 24 animal species for the treatment of over 35 kinds of ailments. The animal species used as traditional medicine by these people consist of fourteen mammals, five birds, three reptiles, one arthropod and one amphibian. Highest number of animal belonged to mammalian taxonomic group (n = 14, 58%), followed by birds (n = 5, 21%) and reptiles (n = 3, 12%). Garasiya people use these animal and their products for the treatment of 35 kinds of different ailments including asthma, paralysis, cough, fever, cold, wound healing etc. These animals were used as whole or by-products of these animals like milk, blood, organ, flesh, tooth, honey, feather etc. for the treatment of various ailments and used in the preparations of traditional medicine [Figure 3, 4, 5, 6].

Fidelity levels (FL) demonstrate the percentage of respondents claiming the use of a certain animals for the same ailments. The uses of animals that are commonly known by the Garasiya informants have higher fidelity level than less common known. The flesh of bat (*Cynopterus sphinx*) used to relieved cough and fever has the highest FL (N = 24, 96%) followed by blood of pigeon (*Columba livia*) to treat paralysis (N = 23, 92%) and urine of cow (*Bos taurus*) for wound healing (N = 23, 92%), while flesh of pig (*Sus scrofa*) to relieved muscular pain (N = 3, 12%) and tooth of elephant (*Elephas maximus*) for pimples (N = 3, 12%) have the lowest FL.

We also mentioned five animal species, used for other religious purposes among these people, theses species were (*Naja naja*, *Panthera tigris, Melursus ursinus, Capra aegagrus, Equus ferus caballus*).

Additional file 1 also shows that cough, asthma, and other respiratory diseases are most frequently cited disease among these people, as such, a number of traditional medicine are available for the treatment of such diseases, many animal by products were used like flesh of frog, honey, milk of goat, and ash of peacock feathers are some of them. Goat (*Capra aegagrus hircus*) and honey bee (*Apis cerana indica*) are most frequently cited animal species among these people, by products of these animals, were used in the treatment of various ailments. Paste of Indian gooseberry + honey used in easily erupting of teeth in child, excreta of crow for the treatment of blister, fur of *lepus *to stop bleeding, were used by Garasiya people in this study area, possibly has been not previously reported in India.

Garasiya people also use one animal product with other animal products or plant derived products to relieve a particular ailment or to prepare traditional medicine, we found that honey is mostly used in many of blend or compound medicine used by Garasiya.

Another important aspect of our study, which needs to be mentioned, is that the Garasiya people also use some endangered, vulnerable and near threatened animal species as medicinal resources. A total of 24 identified animal species, of which 16 (66.65%) are included in the IUCN Red Data list [38]. It is important to mention here that species such as Elephant and Tiger are listed as endangered while Sambhar and Bear are listed as vulnerable and Python is listed as near threatened in IUCN Red Data list. Indian peafowl (the national bird of India) is also listed as vulnerable in the Red Data Book of Indian animals [39]. These tribal people have scarce knowledge, many superstition and myths associated with traditions, which cause harm to animal life. So these traditional medicine and animals by-products should be tested for their appropriate medicinal components, if found indefensible, the people should be aware about the protected and endangered animal species and their importance in biodiversity. Therefore the socio ecological system has to be strengthened through sustainable management and conservation of biodiversity [40] [Table 1].

**Table 1:** Conservation status of animal species (IUCN red list 2009)°

Conservation status	Number of animal species	% of total 24 animal reported
Endangered	02	8.33

Vulnerable	02	8.33

Conservation dependant		

Near threatened	01	4.16

Least concern	11	45.83

Data deficient		

Not evaluated	08	

Total	24	65.65

° source- http://www.iucnredlist.org/ IUCN red list 2009.1

However, with the introduction of the Indian wildlife (protection) Act in 1972, under section 9, hunting of wild animal are strictly prohibited as (*"No person shall hunt any wild animal as specified in schedule I, II, III and IV of the act"*) but sometimes local religious norms in superseded especially in case of saving life. In such cases tribal dare to hunt even the national bird "peacock" [30]. So we would suggest that these kinds of neglected knowledge should be included into the strategies of the conservation and management of faunistic resources.

Despite medicinal purpose, Garasiya also use animal resources for other purpose in their daily life for example, to decorate their traditional houses they are use slough (molted skin of various snake species), this type of decoration are also reported in other communities of India. Ten animal species were also recorded for non-medicinal uses in Kachchh, Gujarat [20]. Moreover they use intestine of goat and hair of horse as wire to making their traditional musical instruments like *Maandal *and *kamayacha*. They also use dried skin of male goat for this purpose. Further studies are required not only to confirm the medicinal value but also to conserve the biodiversity.

## Conclusion

To conclude, a total of 24 animal species were identified for the over 35 kinds of medicinal and other religious purposes used by Garasiya people, inhabitants of village surroundings areas of the Mount Abu wildlife sanctuary, India, out of these 24, five animal species were used for other religious purposes. Mammals consist the highest number of animal (n = 14, 58%) reported for the medicinal purpose. Flesh of bat has the highest FL (96%), while flesh of pig and tooth of elephant have the lowest FL (12%). 66% protected animal species are also mentioned as medicinal resources among these people. Our study also shows that the Garasiya people have very rich folklore and traditional knowledge in the utilization of different animal. So there is an urgent need to properly document to keep a record of the ethnomedicinal data of animal products and their medicinal uses. Further studies are required for scientific validation to confirm medicinal value of such products and to include this knowledge in strategies of conservation and management of animal resources. We hope that this information will be helpful in further research in the field of ethnozoology, ethnopharmacology and biodiversity conservation point of view.

## Competing interests

The authors declare that they have no competing interests.

## Authors' contributions

All authors had significant intellectual contribution towards the design of the field study, data collection, data analysis and write-up of the manuscript. All authors read and approved the final manuscript.

## Supplementary Material

Additional file 1 **List of animal and their products used for traditional medicine by *Garasiya *people of Rajasthan**. The additional file contains information on the medicinal uses of animal and their products in the following pattern: English name, scientific name, and local name of animal species, conservation status of animals according to IUCN red list, part or product or raw material name, ailments, No. of respondents claimed and Fidelity level (FL), mode of preparation and reference to traditional uses in India and other part or location of world. Click here for file
