# Supplementary material for: An ethnozoological study in the adjoining areas of Mount Abu wildlife sanctuary, India
Source: J Ethnobiol Ethnomed. 2010 Feb 10;6:6. doi: 10.1186/1746-4269-6-6 (PMC2836285; doi:10.1186/1746-4269-6-6)
Supplement: Additional file 1 — List of animal and their products used for traditional medicine by Garasiya people of Rajasthan. The additional file contains information on the medicinal uses of animal and their products in the following pattern: English name, scientific name, and local name of animal species, conservation status of animals according to IUCN red list, part or product or raw material name, ailments, No. of respondents claimed and Fidelity level (FL), mode of preparation and reference to traditional uses in India and other part or location of world. [file 1746-4269-6-6-S1.DOC]

**Additional file 1- List of animal and their products used for traditional medicine by *Garasiya* people of Rajasthan.**

| **Animals group** | **Common name** | **Local name** | **Scientific name** | **Conservation status**  ***(IUCN red list 2009) °*** | **Parts or products used** | **Ailments** | **No. of respondents claimed (n)** | **Fidelity level (FL)** | **Mode of preparation** | **Traditional uses in India**  **(reference)** | **Traditional uses in other location**  **(reference)** |
| --- | --- | --- | --- | --- | --- | --- | --- | --- | --- | --- | --- |
| Amphibian | Frog | *Mendak* | *Hoplobatrachus tigerinus*  (Daudin, 1802) | Least Concern | Meat/ flesh | T.B. | 8 | 32% | Cooked meat eaten to cure T.B. and cough. | Whole animal is used for wound [25], flesh and skin were reported [28], skin for wound [40]. | Whole animal in wound [9], flesh for pneumonia [13], body ash use in eyelashes [5]. |
| Arthropods | Honey bee | *Mad- makhi* | *Apis cerana indica*  (Fabricius, 1798) |  | Honey | Facilities in erupting of teeth in child | 16 | 64% | Mixture of Indian gooseberry (*Phyllanthus emblica*) and honey, this paste rubbed on Gums of child. | Honey is used in stomachache and urinary disorder in Attappady hills of western Ghats of India [28]. | Honey is used to cure flu, asthma, thrombosis and TB [9], honey is used in burns, cough [10]. |
| Arthropods | Honey bee | *Mad- makhi* | *Apis cerana indica*  (Fabricius, 1798) |  | Honey | Cough | 20 | 80% | Honey mixed with soup of *adrak* (*Zingiber officinale*) and this paste orally applied thrice times in a day. | Honey is used as eye drop among the inhabitant’s surroundings areas of RNP, and among saharia people of Rajasthan, India [24, 29]. | Honey and wax are used for wound healing, skin diseases, could, fever and cough [5]. |
| Arthropods | Honey bee | *Mad- makhi* | *Apis cerana indica*  (Fabricius, 1798) |  | Honey | Vomiting | 18 | 72% | Solution of honey with water in equal quantity is drunk. |  |  |
| Arthropods | Honey bee | *Mad- makhi* | *Apis cerana indica*  (Fabricius, 1798) |  | Honey | Cough | 22 | 88% | Pure honey is orally drunk at morning and evening. | Honey is also reported for cough, cold [23]. | Honey is used for flu, throat and asthma [14]. |
| Aves | owl | *Gunguraja* | *Otus* *bakkamoena* ([Pennant, 1769](http://www.itis.gov/servlet/SingleRpt/RefRpt?search_type=author&search_id=author_id&search_id_value=24905)) | Least Concern | Wings | Stomach ache | 15 | 60% | Wings burnt and smoke is inhaled. | Flesh of this animal is used as promoter of strength and virility by shoka people [25]. |  |
| Aves | Jungle-fowl | *Murga* | *Gallus sonneratii* (Temminck, 1813) | Least Concern | Whole bird | Influenza | 13 | 52% | Whole animal or flesh fried and eaten to cure influenza. | Blood of domestic hen is used in arthritis [20], raw eggs for prevention of pregnancy, flesh for cough [28], and flesh for asthma [40]. | Fat and gizzard were used to cure asthma, poor digestion and tumor [14], fat of *Gallus* for fever [30]. |
| Aves | Pigeon | *Kabutar* | *Columba livia*  (Gmelin, 1789) | Least Concern | Blood | Paralysis | 23 | 92% | Pure blood is directly applied on affected organ | Flesh for general weakness [22, 40], blood and flesh to cure epilepsy and paralysis [23], same use [20, 29]. |  |
| Aves | Peacock | *Mor* | *Pavo cristatus*  (Linnaeus, 1758) | Least Concern | Leg bone | Ear pain | 22 | 88% | Bone crushed with water and paste is applied. | Same use by saharia, naga and Bhil [22, 29, 34], leg boil with oil in Kachchh [20], Maharashtra [21]. |  |
| Aves | Crow | *Kagla* | *Corvus splendens*  (Vieillot, 1817) | Least Concern | Fecal | Blister  (ulcer on body) | 19 | 76% | Excreta are topically applied. | Flesh is reported for whooping cough [20], anemia [23], leucoderma [28], and paralysis [40]. Bone for ear infection [28], earache [40]. |  |
| Aves | Peacock | *Mor* | *Pavo cristatus*  (Linnaeus, 1758) | Least Concern | Feather | Cough | 16 | 64% | Ash of crown of feather mixed with honey and this paste orally applied. | Feathers are use in infertility [21, 29], flesh for contracted limbs [23], Leg for ear infection [24], and feather’s ash for headache [28], blood for paralysis [30]. |  |
| Mammals | horse | *Ghoda* | *Equus ferus caballus*  (Linnaeus, 1758) |  | Hair of tail | For making musical instruments | 4 | 16% | Hair of tail use as small wire for their musical instruments. | Medicinal use- Flesh of this animal is used for promote strength and eyesight [25], Semen used to treat tetanus and rabbis [29]. | milk and urine are used to treat TB and toothache [5], Wax is used in skin disease [15]. |
| Mammals | Human | *Minakh* | *Homo sapiens sapiens*  (Linnaeus, 1758) |  | Urine | Wound healing/ injury due to cut | 22 | 88% | Directly applied it is an antiseptic. | Same use [22, 24, 29], blood, bone, tooth, nail, hair, saliva are also reported [26], milk and urine are used to relieve eye infection [28]. | Urine and milk are used for skin and eye disease, diphtheria, wound, ear inflammation [5]. |
| Mammals | Elephant | *Hathi* | *Elephas maximus*  (Linnaeus, 1758) | Endangered | Tooth | Pimples on face and beauty | 3 | 12% | Powder of tooth mixed with cream of cow milk and used as cream | Same use in Tamilnadu [23], leucoderma [28], and tooth is used in leucoderma, ringworm, itching and eczema [40]. | Tooth is used to improve women’s fertility [5]. |
| Mammals | Camel | *Unt* | *Camelus dromedarius*  (Linnaeus, 1758) |  | Milk | Acidity | 7 | 28% | Tail of camel is washed in milk and the milk drunk but don’t show this procedure in front of patient. | Blood for arthritis [20], dung is used for constipation [20], milk is used as massage cream [29], and dropping ash is used for stomach ache [30]. | Milk, oil, and urine are used for snake and scorpion bite, liver disease and ulcer in Lavent [5]. |
| Mammals | Pig | *Sur* | *Sus scrofa*  (Linnaeus, 1758) | Least Concern | Flesh | Muscular pain | 3 | 12% | Pure flesh is rubbed on body as cream. | Fat is used for haematoma [23], fat is used to treat muscular pain [24], meat is used as promoter of strength and corpulence [25], fat is applied in paralysis, joints pain, and burns [28]. Ao tribes [40]. | Fat of pig is used in athlete’s foot, Berne, wound healing [14]. |
| Mammals | Bat | *vaagal* | *Cynopterus sphinx*  (Vahl, 1797) | Least Concern | Meat/ flesh | Cough and fever | 24 | 96% | Flesh cooked and then eaten. | Flesh having blood is eaten to treat whooping cough [20], flesh is used in asthma [23] and flesh is also used to treat asthma [40]. | Flesh smoke, blood and brain are used to cure fever, eye disease and to prevent hair growing [5]. |
| Mammals | House Mouse | *Undra* | *Mus musculus*  (Linnaeus, 1758) | Least Concern | Soil of mouse’s burrow | Arthritis | 8 | 32% | Soil mixed with mustard oil and paste is topically use. | Whole animal is used on bald head [20]; meat of this animal is used as promoter of semen [25]. | Body ash is used in inguinal hernias [5]. |
| Mammals | Goat | *Bakri* | *Capra aegagrus hircus*  (Linnaeus, 1758) |  | Intestine  Skin of male goat | To make musical instruments (*maanda*l and *kamayacha*) | 11 | 44% | Dried intestine is use to make small wire and dried skin is use as base, for their musical instruments (*maanda*l and *kamayacha*). | Medicinal use- urine for TB and soup of leg for weakness [29], same use in Kachchh [20], Past of milk and excreta is used in scorpion bite and liver to treat night blindness [30], milk for general weakness [40]. |  |
| Mammals | Goat | *Bakri* | *Capra aegagrus hircus*  (Linnaeus, 1758) |  | Milk | Mouth ulcer and asthma | 21 | 84% | Placing ring around the nipple or teats and than milk stream taken directly in mouth. | Naga [22], urine for TB, milk in eye infection, thoracic vertebra and bone marrow are used in tonsillitis [28]. | Horns is used for ribcage pain [9], horn is used in Israel traditional medicinal system [10, 15]. |
| Mammals | Bear | *Bhalu* | *Melursus ursinus* (Shaw, 1791) | Vulnerable | Tail and claws | Protection from evil | 19 | 76% | Tail and claws are kept in the house to protect from evils. | Medicinal uses- gall bladder is used for curing myriad disease and malaria [25], Fat is used in rheumatism, paralysis and body ache [28], bile is used to treat malaria [40]. |  |
| Mammals | tiger | *Cheeta* | *Panthera tigris* (Linnaeus, 1758) | Endangered | claws | Protection from evil | 22 | 88% | Garasiya people wear claws for the protection from evils. | Same non- medicinal use in Maharashtra [21]. Medicinal uses- flesh and fat are used in leprosy [23], bones for rheumatic and other body pain [27] and fat is used in rheumatism, joint waist pain and burns [28]. | Skin and fur of this animal are also used in Israel traditional medicinal system [15]. |
| Mammals | hare | *Khargosh* | *Lepus nigricollis*  (F. Cuvier, 1823) | Least Concern | Hair/ fur | To stop bleeding | 6 | 24% | Tuft of fur is topically applied on cut area. | Blood is used to treat asthma by shoka [25], liver used in chicken pox [28] and excreta used in ringworm [28]. | Furs are also reported In Nepal [13], and furs of this animal are used in burns [14]. |
| Mammals | Cow | *Gai* | *Bos taurus*  (Linnaeus, 1758) |  | Urine/ saliva | Wound healing | 23 | 92% | Urine or saliva is directly applied on cut area. | Urine is used to cure anemia [30], bile is used in potency, carminative and to promote of auspiciousness [25], and urine is also reported for weakness and cancer, ghee used to neutralizes the snake poison [24]. | Bone marrow, horn, penis are reported for ribcage pain, male impotence, anemia and cough [9], cheese for potency, purgative, eye inflammation, sore throat, burns and cough [10, 15] and fat and bone marrow are also reported for rheumatism and wound healing [14]. |
| Mammals | Monkey | *Vanar* | *Semnopithecus entellus* (Dufresne, 1797) | Least Concern | Meat | rheumatism | 11 | 44% | Cooked flesh is taken | Blood and flesh are reported for asthma [23], flesh is used in asthma [28], and blood is used to treat TB [40]. |  |
| Mammals | Sambar | *Sambar* | *Cervus unicolor* (Kerr, 1792) | Vulnerable | Antlers | stomach ache | 14 | 56% | The paste of antlers is directly applied on stomach. | Penis for hydrocele [23], eye ailments [24], and fat is used in asthma [28] and paste of antler is used in herpes [29]. |  |
| Mammals | cow | *Gai* | *Bos taurus*  (Linnaeus, 1758) |  | Milk | Weakness and for recovery of body power after any disease | 23 | 92% | Pure and fresh milk mixed with turmeric powder and this solution drunk at morning and evening. |  |  |
| Reptiles | Varanus | *Gho* | *Varanus bengalensis* (Daudin, 1802) |  | Flesh/ meat | Improve stamina | 16 | 64% | Cooked flesh is eaten for promote body stamina. | Oil is used for back pain [21], flesh is used in arthritis [23], and fat is used to relieve arthritis [28], and skin, bile duct [40]. |  |
| Reptiles | Snake | *Saanp* | *Naja naja*  (Linnaeus, 1758) |  | Skin (slough) | To decorate home, for worship | 21 | 84% | Slough is used to decorate home and they are used in worship. | Oil of earth boa is applied to treat leucoderma [21], and meat is used to promote eye sight [25]. | Slough is used for skin disease [15]. |
| Reptiles | Python | *Ajgar* | *Python molurus* (Linnaeus, 1758) | Near threatened | Meat/  Skin | Improve eyesight/  Healthy cattle | 4 | 16% | Fried meat is used to improve eyesight while its slough is used for cattle. | Fat is reported for leprosy [23], fat is also reported for rheumatic pain and toothache [28]. |  |

**° source- (**[**http://www.iucnredlist.org/**](http://www.iucnredlist.org/)**) IUCN red list 2009.1**
